# Efficacy and safety of red ginseng extract powder (KGC05pg) in achieving glycemic control in prediabetic Korean adults: A 12-week, single-center, randomized, double-blind, parallel-group, placebo-controlled study

**DOI:** 10.1097/MD.0000000000041130

**Published:** 2024-12-27

**Authors:** Yoonseon Jeong, Seung Ho Lee, Sung Lye Shim, Kyoung Hwa Jang, Jong Han Kim

**Affiliations:** a Korea Ginseng Corporation Research Institute, Gwacheon, Gyeonggi, Republic of Korea.

**Keywords:** diabetes mellitus, glycemic control, insulin, insulin resistance, panax

## Abstract

**Background:**

This study was conducted to assess the efficacy and safety of Red Ginseng Extract Powder (RGEP) (KGC05pg; Korea Ginseng Corporation, Daejeon, Korea) in achieving glycemic control in prediabetic Korean adults.

**Methods:**

The participants of the RGEP group (n = 49) and those of the placebo group (n = 49) were orally given 2 tablets of RGEP and its matching placebo, respectively, at a dose of 500 mg/day twice daily in the morning and the evening within 30 min after meal during a 12-week study period. The participants were assessed for glycemic control parameters, such as fasting blood glucose levels, 30-, 60-, 90-, and 120-min blood glucose levels on an oral glucose tolerance test, Hb1Ac levels and glucose area under the curve, insulin resistance parameters, such as homeostasis model assessment of insulin resistance, c-peptide and insulinogenic index, and hormone parameters, such as glucagon, adiponectin and glucagon-like peptide-1. Moreover, the participants were also assessed for time-dependent changes in dipeptidyl peptidase-4 levels. Finally, the participants were also assessed for incidences of treatment-emergent adverse events and serious adverse events.

**Results:**

There were significant differences in changes in fasting blood glucose and 30-, 60-, 90-, and 120-min blood glucose levels on an oral glucose tolerance test, Hb1Ac levels, glucose area under the curve, homeostasis model assessment of insulin resistance, c-peptide levels and insulinogenic index, glucagon, adiponectin, and glucagon-like peptide-1 levels at 12 weeks from baseline between the 2 groups (*P* < .05). There was a significant time-dependent decrease in dipeptidyl peptidase-4 levels in the RGEP group (*P* = .001). There were no cases of treatment-emergent adverse events and serious adverse events in each study group.

**Conclusion:**

RGEP might be effective in achieving glycemic control in prediabetic Korean adults.

## 1. Introduction

With the continuous globalization of modern society, resulting in changes in dietary and lifestyle habits, there has been an increase in the prevalence of chronic diseases. Of these, the prevalence of diabetes mellitus (DM) is rapidly increasing worldwide.^[1]^ According to a survey performed by the International Diabetes Federation, as of 2021, there are approximately 537 million people with diabetes worldwide (a prevalence rate of 9.8%), around 319 million people with impaired fasting glucose (a prevalence rate of 5.7%), and approximately 541 million people with impaired glucose tolerance (a prevalence rate of 10.2%).^[2]^ These numbers are expected to continue rising. According to the Korean Diabetes Association Fact Sheet 2020, in 2018, the prevalence of DM among adults over 30 years of age was 13.8% (about 4.94 million people), indicating that 1 in every 7 adults over the age of 30 has it. The prevalence of impaired fasting glucose was also estimated to be 26.9% (about 9.48 million people).^[3]^

Korean red ginseng is made by steaming unpeeled ginseng (fresh ginseng) and then drying it. Korean red ginseng has a unique aroma and taste; it is slightly sweet at first and slightly bitter afterward, with a warm property.^[4,5]^ Previous literatures have described its health benefits. First, it is recorded as protecting the 5 viscera (liver, heart, lung, kidney, and spleen), stabilizing the mind, brightening the eyes, and promoting longevity with long-term use. Second, it restores physical weakness, assists in the functions of the 5 viscera and 6 bowels and has effects on the symptoms of DM, such as generating body fluids and relieving thirst. In addition to these effects, it is known to improve memory and learning ability, enhance immunity, have anticancer effects, alleviate fatigue and stress, improve blood circulation and treat anemia, have antiaging effects, relieve hangovers, prevent osteoporosis, treat male infertility and improve hypertension and diabetes.^[6–8]^

Given the above background, red ginseng extract powder (RGEP) (KGC05pg; Korea Ginseng Corporation, Daejeon, Korea), a safe natural substance, has been prepared to improve glycemic control.^[9]^ This study was conducted to assess its efficacy and safety in achieving glycemic control in prediabetic Korean adults.

## 2. Materials and methods

### 2.1. Study participants and setting

The current 12-week, single-center, randomized, double-blind, parallel-group, placebo-controlled study was conducted at Seoul National University Bundang Hospital in Korea between April 3 and October 23, 2023.

Inclusion criteria for the current study are as follows:

1. Korean men or women aged ≥19 years old.
2. The participants with fasting blood glucose (FBG) 100 to 125 mg/dL or postprandial glucose (PPG) 140 to 199 mg/dL who had no past history of taking antihyperglycemic drugs.
3. The participants who were informed of details of the current study and submitted a written informed consent for study participation.

Major exclusion criteria for the current study are as follows:

1. The participants who were given drugs, such as oral hypoglycemic drugs (e.g., sulfonylurea, biguanides, α-glucosidase inhibitor, meglitinides, thiazolidinediones, dipeptidyl peptidase [DPP]-4 inhibitor, sodium glucose cotransporter-2 inhibitor, injectable hypoglycemic agents [e.g., glucagon-like peptide-1 {GLP-1} receptor agonist]), insulin therapy, or health functional foods, RGEP for the purposes of controlling blood glucose levels before study entry.
2. The participants with a past history of type I or II DM or pregnancy DM (A2 or B1).
3. The participants with FBG ≥ 126 mg/dL or PPG ≥ 200 mg/dL.
4. The participants with HbA1c ≥ 6.5%.
5. The participants with a past history of taking diuretics, β-blockers, anticoagulants and antiplatelet agents.
6. The participants with a past history of coronary artery disease, myocardial infarction, angina pectoris, heart failure, ischemic heart disease, atherosclerosis, arrhythmia, cerebral infarction, stroke, pulmonary disease, systemic autoimmune disease, rheumatoid arthritis, thyroid disease and hypertension.
7. The participants who were given drugs, such as anti-obesity agents (e.g., GLP-1 receptor agonist, absorption inhibitor and appetite suppressant) and anti-dyslipidemic agent, or health functional foods for the purposes of improving the obesity and lipid profile before study entry.

The current study was approved by the Institutional Review Board of Seoul National University Bundang Hospital in Korea (Institutional Review Board approval # B-2301-806-001) and then conducted in compliance with the relevant ethics guidelines. All the study procedures described herein were performed in accordance with the 1964 Declaration of Helsinki and its later amendments or comparable ethical standards. The current study was conducted in compliance with the International Conference of Harmonization-Good Clinical Practice and it is registered with the Clinical Research information Service (KCT0009340). All the participants submitted a written informed consent for study participation.

### 2.2. Rationale of sample size estimation

Ranasinghe P, et al conducted a randomized double-blind placebo-controlled clinical trial in a total of 200 subjects. According to these authors, the FBG levels were measured as 144.9 ± 28.8 mg/dL in the RGEP group and 162.9 ± 22.4 mg/dL in the placebo group after zinc supplementation.^[10]^ Based on this previous published trial, both inter-group differences and variance were estimated for the current study. Considering a drop-out rate of 10%, the minimum number of the participants per group was calculated as 44. Therefore, the final number of the participants per group was estimated at 49 based on a formula 44/(1−0.1). It was planned that a total of 98 participants would be enrolled in the current study.

### 2.3. Trial methods

**Figure 1.**
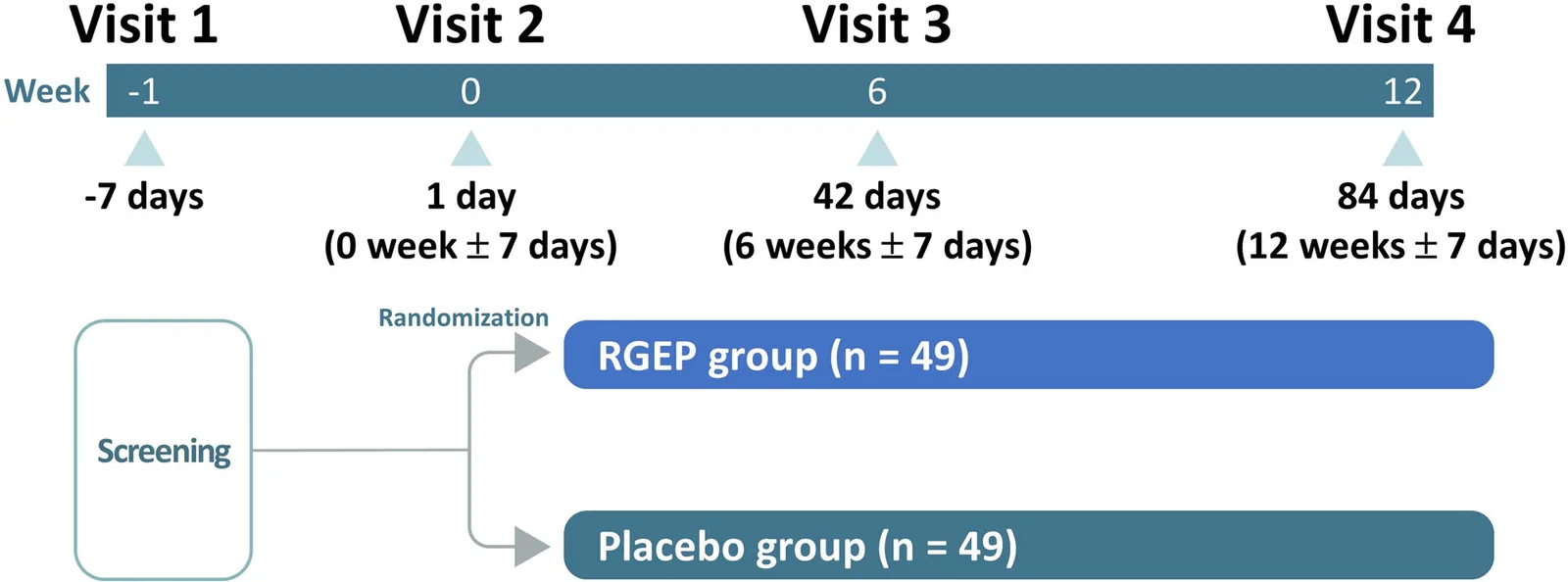
Study design.

Once determined to be eligible for study participation, the participants were evaluated for clinical examinations at baseline. At Visit 2, they were randomized either to the RGEP group or the placebo group, followed by a 12-week supplementation period. The study schema is illustrated in Figure 1.

The participants of the RGEP group were orally given 2 tablets of RGEP at a dose of 500 mg/day twice daily in the morning and the evening within 30 min after meal during a 12-week supplementation period. The participants of the placebo group were also orally given 2 tablets of its matching placebo at a dose of 500 mg/day twice daily in the morning and the evening within 30 minutes after meal during a 12-week supplementation period.

### 2.4. Randomization and blinding

The randomization table was provided by the contract research organization, based on which the participants were randomized to either of two study groups using a block randomization. To do this, according to the uniform distribution, computer-generated lists of random numbers were created using Random Number Generators of SPSS software (IBM Corp., Armonk, NY). At the time of study entry, the principal investigator was provided with a randomization envelope from the contract research organization. This is followed by allocation of random numbers to all the eligible participants according to the order of study products.

The participants of each study group were provided with study products whose specification and formulation are identical. Therefore, both the principal investigator and the participants would be unaware of allocation of a certain participants to a specific study group unless a randomization envelope is opened. Thus, the double-blind status was maintained until study period have been completed.

### 2.5. Assessment criteria and outcome measures

Baseline characteristics of the participants include age, height, weight, body mass index, smoking status, drinking status, and exercise.

For efficacy assessment, the participants were assessed for glycemic control parameters, such as FBG levels, 30-, 60-, 90-, and 120-min blood glucose levels on an oral glucose tolerance test (OGTT), Hb1Ac levels and glucose area under the curve, insulin resistance parameters, such as homeostasis model assessment of insulin resistance, c-peptide and insulinogenic index, and hormone parameters, such as glucagon, adiponectin, and GLP-1. Moreover, the participants were also assessed for time-dependent changes in DPP-4 levels.

Both full analysis and per-protocol analysis were performed, for which full analysis set and per-protocol set were provided.

For safety assessment, any adverse events (AEs) were categorized by the system organ class and then coded by preferred terms using the Medical Dictionary for Regulatory Activities version 19, as previously described.^[11]^ Then, the participants were assessed for incidences of treatment-emergent AEs and serious AEs.

### 2.6. Statistical analysis

Measurements were presented as mean ± standard deviation or the number of the participants with percentage, where appropriate. In each study group, changes in outcome measures at 12 weeks from baseline were analyzed using the Wilcoxon signed-rank test. In addition, efficacy and safety outcome measures were analyzed using independent two-sample *t* test or Wilcoxon rank sum test, if applicable. Statistical analysis was done using the Statistical Analysis Software Version 9.4 (SAS Institute Inc, Cary, NC). Statistical significance was set at *P* < .05.

## 3. Results

### 3.1. Baseline characteristics of the participants

**Figure 2.**
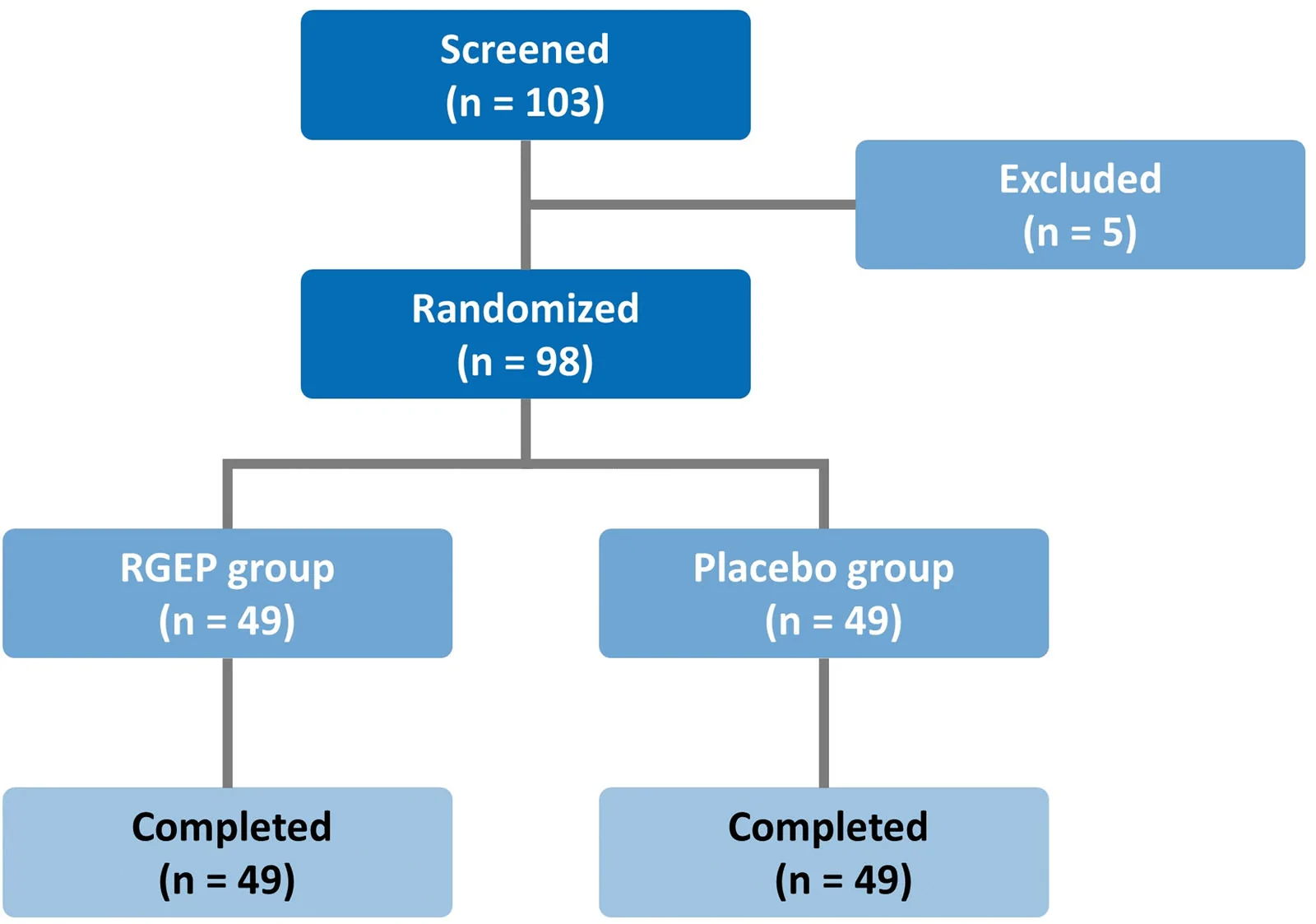
Disposition of the study participants.

A total of 98 participants (n = 98) were finally enrolled in the current study, who were equally randomized to the RGEP group (n = 49) and the placebo group (n = 49). The disposition of the study participants is illustrated in Figure 2.

There were no significant differences in age, sex, height, weight, body mass index, smoking status, drinking status, and exercise between the 2 groups (*P* > .05) (Table 1).

**Table 1:** Baseline characteristics of the participants (n = 98).

Variables	Values		*P*-value
	RGEP group (n = 49)	Placebo group (n = 49)	
Age (years old)	61.33 ± 11.54 (38–78)	59.22 ± 14.22 (24–80)	.424
*Sex*			
Men	24 (49.0%)	26 (53.1%)	.686
Women	25 (51.0%)	23 (46.9%)	
Height (cm)	162.32 ± 8.47	164.45 ± 7.89	.202
Weight (kg)	65.24 ± 9.25	67.35 ± 9.90	.279
BMI (kg/m^2^)	24.69 ± 2.26	24.83 ± 2.49	.767
*Smoking status*			
Never	29 (59.2%)	27 (55.1%)	.913
Past smoker	6 (12.2%)	7 (14.3%)	
Current smoker	14 (28.6%)	15 (30.6%)	
*Drinking status*			
Never	22 (44.9%)	18 (36.7%)	.492
Past drinker	3 (6.1%)	6 (12.2%)	
Current drinker	24 (49.0%)	25 (51.0%)	
*Exercise*			
No	20 (40.8%)	23 (46.9%)	.541
Yes	29 (59.2%)	26 (53.1%)	

Values are mean ± standard deviation with the range or the number of the participants with percentage, where appropriate.

BMI = body mass index, RGEP = red ginseng extract powder.

### 3.2. Efficacy outcomes

In the current study, there were no participants who were ineligible for the per-protocol analysis; there were no participants who were dropped out of the current study, used prohibited concomitant drugs or foods, had a ≤80% compliance with study products or seriously violated the protocol. There was no difference in the number of the participants between the full analysis set and the per-protocol set. Therefore, the full analysis was solely performed in the current study.

**Figure 3.**
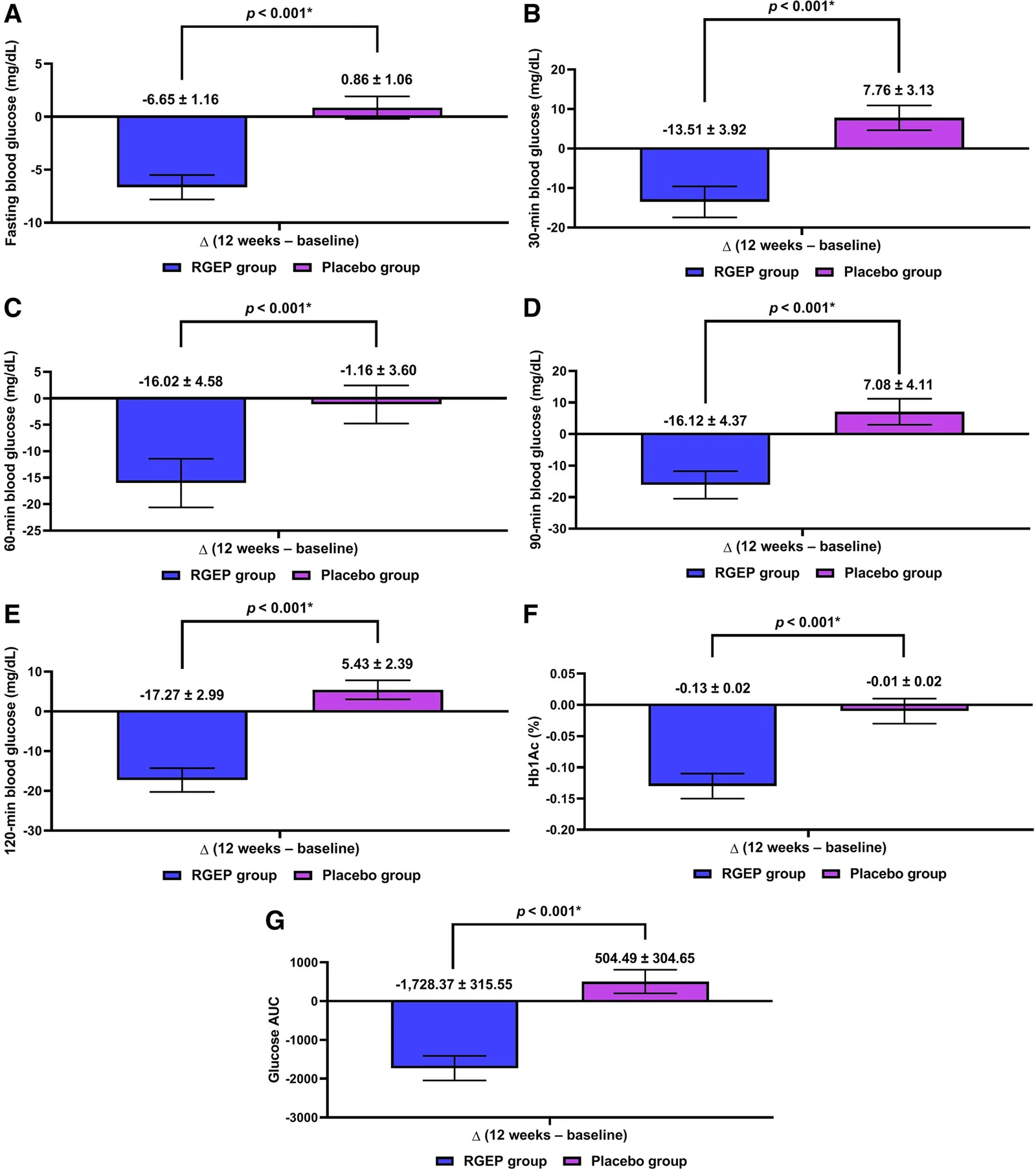
Differences in changes in glycemic control parameters at 12 weeks from baseline between the 2 groups. (A) Fasting blood glucose, (B) 30-min blood glucose, (C) 60-min blood glucose, (D) 90-min blood glucose, (E) 120-min blood glucose, (F) Hb1Ac, and (G) glucose AUC. AUC = area under the curve. Values are mean ± standard deviation. *Statistical significance at *P* < .05.

Time-dependent changes in glycemic control parameters are represented in Table 2; there were significant differences in changes in FBG and 30-, 60-, 90-, and 120-min blood glucose levels on an OGTT, Hb1Ac levels and glucose area under the curve at 12 weeks from baseline between the 2 groups (*P* < .05) (Fig. 3).

**Table 2:** Time-dependent changes in glycemic control parameters (n = 98).

Variables	Values	
	RGEP group (n = 49)	Placebo group (n = 49)
*Fasting blood glucose (mg/dL*)		
Baseline	107.55 ± 6.64	106.45 ± 4.80
6 weeks	105.51 ± 8.07	106.63 ± 7.74
12 weeks	100.90 ± 9.46	107.31 ± 7.62
*30-min blood glucose (mg/dL*)		
Baseline	173.51 ± 25.34	168.20 ± 17.56
6 weeks	168.65 ± 20.70	173.92 ± 27.01
12 weeks	160.00 ± 19.49	175.96 ± 26.68
*60-min blood glucose (mg/dL*)		
Baseline	226.76 ± 30.05	223.06 ± 25.03
6 weeks	223.90 ± 32.04	219.16 ± 35.68
12 weeks	210.73 ± 27.47	221.90 ± 35.38
*90-min blood glucose (mg/dL*)		
Baseline	200.49 ± 23.00	194.08 ± 18.43
6 weeks	197.53 ± 36.41	200.22 ± 31.03
12 weeks	184.37 ± 30.37	201.16 ± 33.77
*120-min blood glucose (mg/dL*)		
Baseline	170.53 ± 15.30	167.33 ± 13.73
6 weeks	163.86 ± 19.38	170.27 ± 20.88
12 weeks	153.27 ± 22.43	172.76 ± 22.24
*Hb1Ac levels (%*)		
Baseline	5.98 ± 0.18	5.96 ± 0.14
6 weeks	5.95 ± 0.18	5.95 ± 0.18
12 weeks	5.85 ± 0.18	5.95 ± 0.16
*Glucose AUC*		
Baseline	22,100.63 ± 1.10	21,596.89 ± 1.08
6 weeks	21,618.36 ± 1.11	21,793.50 ± 1.13
12 weeks	20,370.90 ± 1.10	21,996.44 ± 1.13

Values are mean ± standard deviation.

AUC = area under the curve, RGEP = red ginseng extract powder.

**Figure 4.**
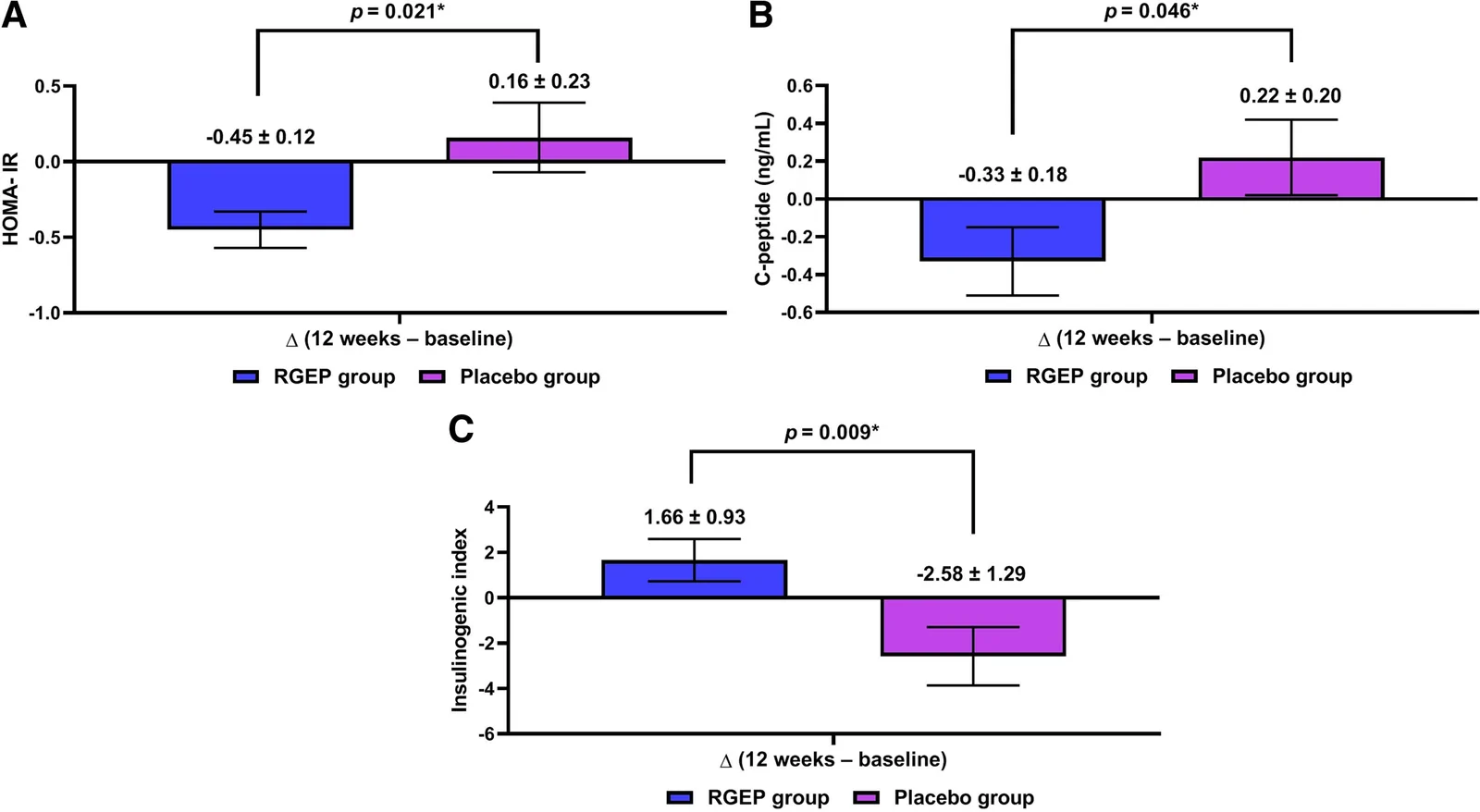
Differences in changes in insulin resistance parameters at 12 weeks from baseline between the 2 groups. (A) HOMA-IR, (B) C-peptide, and (C) insulinogenic index. HOMA-IR = homeostasis model assessment of insulin resistance. Values are mean ± standard deviation. *Statistical significance at *P* < .05.

Time-dependent changes in insulin resistance parameters are represented in Table 3; there were significant differences in changes in homeostasis model assessment of insulin resistance, c-peptide levels and insulinogenic index at 12 weeks from baseline between the 2 groups (*P* < .05) (Fig. 4).

**Table 3:** Time-dependent changes in insulin resistance parameters (n = 98).

Variables	Values	
	RGEP group (n = 49)	Placebo group (n = 49)
*HOMA-IR*		
Baseline	2.26 ± 1.53	2.24 ± 1.68
6 weeks	2.04 ± 1.78	2.26 ± 1.85
12 weeks	1.82 ± 1.57	2.36 ± 1.71
*C-peptide (ng/mL*)		
Baseline	2.31 ± 1.58	2.19 ± 1.58
6 weeks	2.15 ± 1.67	2.64 ± 1.71
12 weeks	2.11 ± 1.50	2.40 ± 1.56
*Insulinogenic index*		
Baseline	5.32 ± 2.38	5.45 ± 2.72
6 weeks	6.61 ± 2.77	5.33 ± 2.35
12 weeks	7.19 ± 2.02	4.94 ± 2.20

Values are mean ± standard deviation.

HOMA-IR = homeostasis model assessment of insulin resistance, RGEP = red ginseng extract powder.

**Figure 5.**
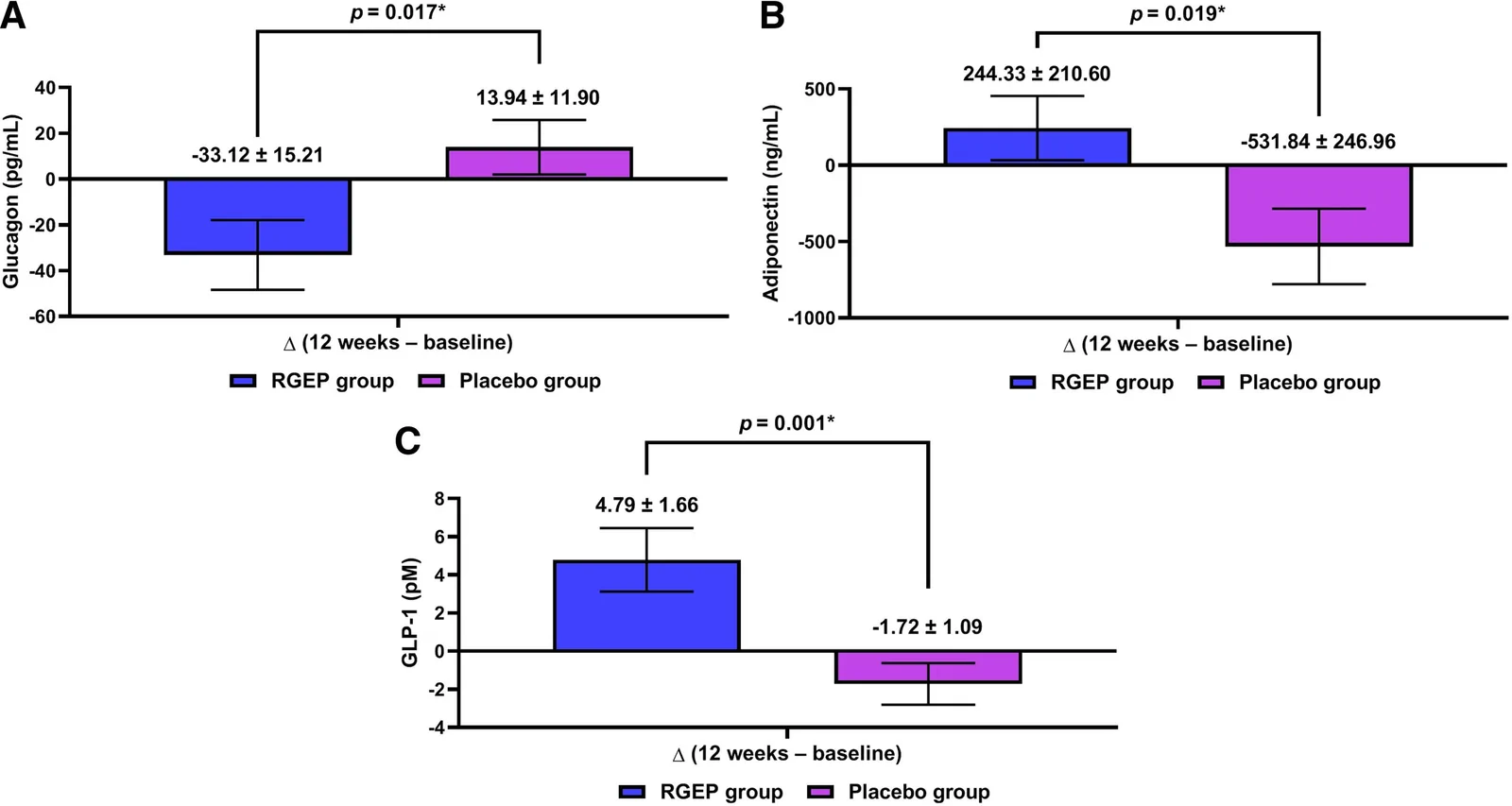
Differences in changes in hormone parameters at 12 weeks from baseline between the 2 groups. (A) Glucagon, (B) adiponectin, and (C) GLP-1. GLP-1 = glucagon-like peptide-1. Values are mean ± standard deviation. *Statistical significance at *P* < .05.

Time-dependent changes in hormone parameters are represented in Table 4; there were significant differences in changes in glucagon, adiponectin and GLP-1 levels at 12 weeks from baseline between the 2 groups (*P* < .05) (Fig. 5).

**Table 4:** Time-dependent changes in hormone parameters (n = 98).

Variables	Values	
	RGEP group (n = 49)	Placebo group (n = 49)
*Glucagon (pg/mL*)		
Baseline	178.53 ± 1.57	127.30 ± 1.75
6 weeks	170.58 ± 1.56	153.94 ± 1.50
12 weeks	150.23 ± 1.49	143.23 ± 1.65
*Adiponectin (ng/mL*)		
Baseline	5281.11 ± 1.85	5643.86 ± 1.84
6 weeks	5571.20 ± 1.82	5292.17 ± 1.81
12 weeks	5559.13 ± 1.80	5208.91 ± 1.81
*GLP-1 (pM*)		
Baseline	26.62 ± 1.44	29.83 ± 1.36
6 weeks	27.10 ± 1.46	26.27 ± 1.39
12 weeks	30.38 ± 1.55	27.65 ± 1.44

Values are mean ± standard deviation.

GLP-1 = glucagon-like peptide-1, RGEP = red ginseng extract powder.

**Figure 6.**
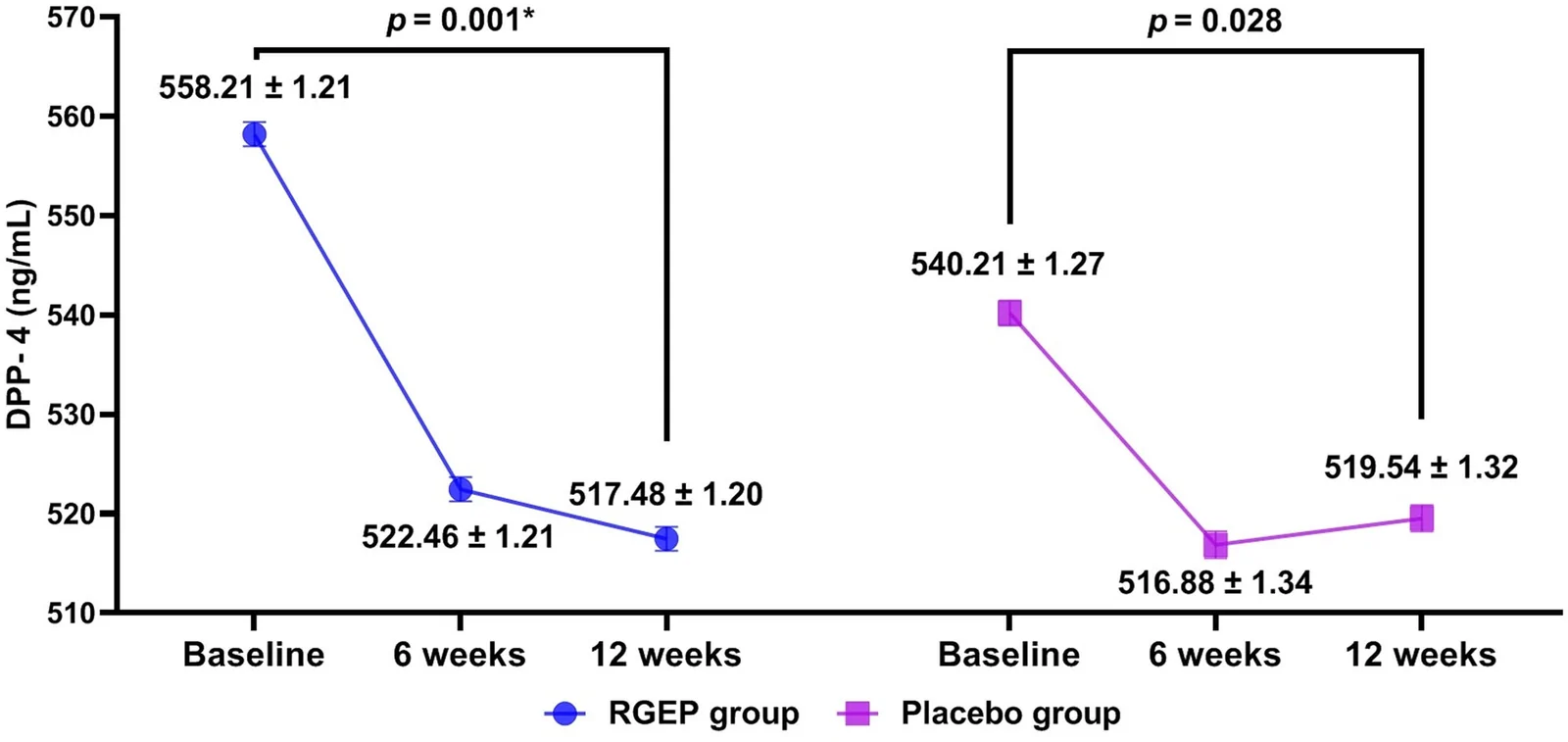
Time-dependent changes in dipeptidyl peptidase-4 (DPP-4) levels in each group. Values are mean ± standard deviation. *Statistical significance at *P* < .05.

Of note, there was a significant time-dependent decrease in DPP-4 levels in the RGEP group (*P* = .001) (Fig. 6).

### 3.3. Safety outcomes

There were no cases of treatment-emergent AEs and serious AEs in each study group.

## 4. Discussion

DM is referred to as a condition where glucose, serving as the energy source for human body, is not used for energy and remains in the bloodstream, eventually being excreted in the urine.^[12]^ Its diagnostic criteria include FBG of ≥126 mg/dL and PPG of ≥200 mg/dL or Hb1Ac levels of ≥6.5%.^[13]^ The prediabetic stage is characterized by higher blood glucose levels as compared with normal but lower as compared with diagnostic criteria for DM.^[14,15]^ Impaired fasting glucose is characterized by the FBG levels of 100 to 125 mg/dL after an 8-hour fast. Moreover, impaired glucose tolerance is defined as blood glucose levels of 140 to 199 mg/dL at 2 hours after an OGTT.^[16]^

Primary symptoms of DM include polydipsia, polyphagia and polyuria. Other potential symptoms include blurred vision, itching of the skin or genital area and weight loss.^[17]^ Lack of notable symptoms may lead to a delayed diagnosis of DM. If prolonged, DM would cause complications, such as cardiovascular diseases, decreased vision or blindness, insufficient renal functions, neural fatigue, seizures, and ulcers on the limbs. But the prediabetic stage is often characterized by lack of notable symptoms. Increased insulin resistance with hyperinsulinemia, excessive insulin secretion as a compensatory response may raise a risk of developing DM and its cardiovascular diseases. This highlights the importance of prevention during the prediabetic stage.^[18–21]^

Treatment options for DM include smoking cessation, abstinence of alcohol consumption, regular aerobic and resistance exercises, balanced dietary intake and consistent self-monitoring of blood glucose.^[22,23]^ Medication options include oral hypoglycemics (sulfonylureas, biguanides, α-glucosidase inhibitors, meglitinides, thiazolidinediones, DPP-4 inhibitors, and sodium glucose cotransporter-2 inhibitors) and insulin therapy.^[24]^ It is unavoidable, however, that medication may cause diverse adverse effects, such as gastrointestinal problems, hypoglycemia, edema, weight gain, severe abdominal pain, liver dysfunction, anemia, joint pain, and urinary tract infections.^[25]^ During the prediabetic stage, exercise, and dietary interventions are recommended over specific treatments, although the practical application and adherence to these recommendations can be challenging, often leading to exclusion from treatment considerations.^[26,27]^

To minimize the adverse effects of pharmacotherapy and to improve blood glucose levels, ongoing studies are conducted. Thus, attempts are made to discover bioactive substances from natural products. The Korean Ministry of Food and Drug Safety has recognized several ingredients for their functional properties.^[28]^ These functional ingredients have been recognized for their potential health benefits, including their ability to help manage blood glucose levels. These functional ingredients have been recognized for their potential health benefits, including their ability to help manage blood glucose levels. There is a growing trend toward using natural and functional foods to support health and well-being, particularly in the prevention and treatment of conditions like DM.^[29,30]^ From this context, potential health benefits of RGEP deserve special attention; its raw material is registered with Food Standards and Specifications No. 2022-76, dated October 25, 2022, Standard and Specifications for Health Functional Foods No. 2022-66, dated September 15, 2022 and the Korean Pharmacopoeia (Notification No. 2018-16, dated March 8, 2018). It is also listed in overseas pharmacopoeias, such as the United Natural Products Alliance and the American Herbal Products Association in the United States, Health Canada and Chinese pharmacopoeias. Traditionally, it has been widely used in Korean traditional medicine prescriptions and is still sold in various forms. The safety of the raw material has been confirmed through domestic standards and specifications for pharmaceuticals and food, as well as standards and specifications for functional health foods.^[7]^ Moreover, antihyperglycemic effects of RGEP have been widely explored. That is, preclinical testing has been conducted, confirming significant improvements in indicators of insulin resistance (pAMPK and pACC) and fatty acid oxidation rates in vitro, as well as improvements in FBG, postprandial blood glucose, HbA1c, indicators of renal function, abnormal lipidemia, and inflammation-related indicators in vivo.^[31,32]^

The current study showed that RGEP was effective in significantly improving FBG levels, 30-, 60-, 90-, and 120-min blood glucose levels and Hb1Ac levels (*P* < .05). This is in agreement with previous published studies. Antihyperglycemic effects of RGEP have been well described in the literature; it has been used as a complementary and alternative medicine for better glycemic control among individuals with type II DM.^[33]^ Its efficacy in improving PPG and insulin sensitivity has been documented.^[34,35]^ Presumably, antihyperglycemic effects of RGEP might arise from its anti-inflammatory and anti-oxidative properties.^[36–39]^ This has been well described in previous literatures. According to Park JK, et al, there were significant improvements in FBG, HbA1c, inflammatory markers (e.g., interleukin-6, cyclooxygenase-2, and C-reactive protein) following a 12-week administration of RGEP100 mg/kg in db/db mice.^[39]^ Kang KS, et al showed that Rg3 was effective in protecting against diabetes by inhibiting oxidative stress in a streptozotocin-induced diabetic renal damage model.^[40]^ Finally, Rg3 had an antihyperglycemic effect by up-regulating GLP-1 *via* the sweet taste receptor-mediated signal transduction pathway in db/db mice.^[41]^

The current study also showed that RGEP was effective in significantly improving insulin resistance (*P* < .05). The efficacy of RGEP in improving insulin resistance has been reported to have a relationship with adipocyte hypertrophy; adipocyte hypertrophy and atrophy are associated with insulin resistance and insulin sensitivity, respectively.^[42]^ It has been shown that ginsenoside Rg3, linked to STAT5-PPARγ pathway, was effective in improving obesity-induced insulin resistance.^[43]^ Taken together, the efficacy of RGEP in improving insulin resistance arises from its inhibitory effect against adipocyte hypertrophy in obese animals. Hossain MA, et al showed that RGEP was effective in significantly improving hyperglycemia and oxidative stress in Otsuka Long-Evans Tokushima fatty rats.^[44]^

In the current study, there was a significant time-dependent decrease in DPP-4 levels in the RGEP group (*P* = .001). This is in agreement with a previous published study.^[45]^ Of diverse active constituents of RGEP, alkaloids play a role in inhibiting the activity of DPP-4 that degrades the GLP-1 hormone.^[46]^ This was also seen in a previous report showing that alkaloids play a role in inhibiting DPP-4, advanced glycation end products, increment of insulin release and secretion from pancreatic β-cell and its sensitivity and the enhancement of glucose uptake from circulation to lower the levels.^[47]^ A significant time-dependent decrease in DPP-4 levels following the supplementation with RGEP suggests that alkaloids act as a DPP-4 inhibitor by preventing DPP-4 from binding to and interacting with the GLP-1.

To summarize, the current results are as follows:

1. There were significant differences in changes in FBG and 30-, 60-, 90-, and 120-min blood glucose levels on an OGTT, Hb1Ac levels, and glucose area under the curve at 12 weeks from baseline between the 2 groups (*P* < .05).
2. There were significant differences in changes in homeostasis model assessment of insulin resistance, c-peptide levels and insulinogenic index at 12 weeks from baseline between the 2 groups (*P* < .05).
3. There were significant differences in changes in glucagon, adiponectin and GLP-1 levels at 12 weeks from baseline between the 2 groups (*P* < .05).
4. There was a significant time-dependent decrease in DPP-4 levels in the RGEP group (*P* = .001).
5. There were no cases of treatment-emergent AEs and serious AEs in each study group.

But the current results cannot be generalized; there are several limitations of the current study as follows: First, this is a small-scale study. Second, the possibility of selection bias could not be completely ruled out because this is a single-center study. Single-center studies are often followed by multicenter ones.^[48]^ A collaborative, multicenter is equipped with key advantages, such as an ability to respond to questions requiring a larger sample size and those about the generalizability of outcomes across the centers and to compare the effects between the study centers.^[49,50]^ Further large-scale, multicenter studies are therefore warranted to corroborate the current results. Third, the current study did not assess whether RGEP was effective in lowering a risk of diabetic complications that are mediated by chronic hyperglycemia with consequent oxidative stress, advanced glycation end products, and diverse inflammatory responses. According to animal studies, however, RGEP can reduce the occurrence of diabetic microvascular complications and brain neuronal damage by inhibiting oxidative stress and advanced glycation end product.^[51–53]^ This also warrants further clinical trials.

## 5. Conclusion

Based on the current results, it can be concluded that RGEP might be effective in achieving glycemic control in prediabetic Korean adults. But this warrants further large-scale, multicenter, prospective, randomized, controlled studies.

## Acknowledgements

The current study was sponsored by Korea Ginseng Corporation (KGC-ER 23-0061). All the authors of the current study are employees of Korea Ginseng Corporation, who have no relevant affiliations or financial involvement with any organization or entity with a financial interest in or financial conflict with drugs or medical equipments or devices mentioned in the current study.

## Author contributions

**Conceptualization:** Yoonseon Jeong.

**Data curation:** Yoonseon Jeong.

**Formal analysis:** Yoonseon Jeong.

**Funding acquisition:** Sung Lye Shim.

**Investigation:** Seung Ho Lee.

**Methodology:** Seung Ho Lee.

**Project administration:** Sung Lye Shim.

**Resources:** Kyoung Hwa Jang.

**Software:** Kyoung Hwa Jang.

**Supervision:** Kyoung Hwa Jang.

**Validation:** Sung Lye Shim.

**Visualization:** Sung Lye Shim.

**Writing – original draft:** Yoonseon Jeong, Jong Han Kim.

**Writing – review & editing:** Kyoung Hwa Jang, Jong Han Kim.

## Correction

This article was originally published using the terms "Patient(s), “treatment period,” “treatment arm,” “treatment group,” and "treatment." These terms have now been updated in the online version with “Participant(s),” “supplementation period,” “study group,” and “supplementation” appropriately.

## References

[1] Hu FB. Globalization of diabetes: the role of diet, lifestyle, and genes. Diabetes Care. 2011;34:1249–57. 21617109 10.2337/dc11-0442 PMC3114340

[2] International Diabetes Federation. IDF Diabetes Atlas 10th edition. Available at: https://diabetesatlas.org/idfawp/resource-files/2021/07/IDF_Atlas_10th_Edition_2021.pdf [access date March 25, 2024].

[3] Jung CH Son JW Kang S. Diabetes fact sheets in Korea, 2020: an appraisal of current status. Diabetes Metab J. 2021;45:1–10. 33434426 10.4093/dmj.2020.0254 PMC7850879

[4] Hyun SH Ahn HY Kim HJ. Immuno-enhancement effects of Korean Red Ginseng in healthy adults: a randomized, double-blind, placebo-controlled trial. J Ginseng Res. 2021;45:191–8. 33437171 10.1016/j.jgr.2020.08.003 PMC7790881

[5] Lee SM Bae BS Park HW. Characterization of Korean red ginseng (panax ginseng meyer): history, preparation method, and chemical composition. J Ginseng Res. 2015;39:384–91. 26869832 10.1016/j.jgr.2015.04.009 PMC4593794

[6] Panossian A Abdelfatah S Efferth T. Network pharmacology of ginseng (part III): antitumor potential of a fixed combination of red ginseng and red sage as determined by transcriptomics. Pharmaceuticals (Basel). 2022;15:1345. 36355517 10.3390/ph15111345 PMC9696821

[7] So SH Lee JW Kim YS Hyun SH Han C-K. Red ginseng monograph. J Ginseng Res. 2018;42:549–61. 30337816 10.1016/j.jgr.2018.05.002 PMC6190493

[8] Park SK Kim SW Seo HW. Long-term evaluation of safety and biological effects of Korean Red Ginseng (Panax Ginseng): a long-term in vivo study. BMC Complement Med Ther. 2022;22:284. 36333693 10.1186/s12906-022-03736-5 PMC9635099

[9] Lee SY. A scientific evidence showing glycemic control effects of red ginseng extract powder. Available at: https://www.edaily.co.kr/News/Read?newsId=02361606639088016&mediaCodeNo=257 [access date November 24, 2024].

[10] Ranasinghe P Wathurapatha WS Galappatthy P Katulanda P Jayawardena R Constantine GR. Zinc supplementation in prediabetes: a randomized double-blind placebo-controlled clinical trial. J Diabetes. 2018;10:386–97. 29072815 10.1111/1753-0407.12621

[11] Yakoot M Abdo AM Abdel-Rehim S Helmy S. Response tailored protocol versus the fixed 12weeks course of dual sofosbuvir/daclatasvir treatment in Egyptian patients with chronic hepatitis C genotype-4 infection: a randomized, open-label, non-inferiority trial. EBioMedicine. 2017;21:182–7. 28647541 10.1016/j.ebiom.2017.05.011 PMC5514382

[12] Chadt A Al-Hasani H. Glucose transporters in adipose tissue, liver, and skeletal muscle in metabolic health and disease. Pflugers Arch. 2020;472:1273–98. 32591906 10.1007/s00424-020-02417-x PMC7462924

[13] Ghazanfari Z Haghdoost AA Alizadeh SM Atapour J Zolala F. A comparison of HbA1c and fasting blood sugar tests in general population. Int J Prev Med. 2010;1:187–94. 21566790 PMC3075530

[14] Di Pino A Urbano F Piro S Purrello F Rabuazzo AM. Update on pre-diabetes: focus on diagnostic criteria and cardiovascular risk. World J Diabetes. 2016;7:423–32. 27795816 10.4239/wjd.v7.i18.423 PMC5065662

[15] Khan RMM Chua ZJY Tan JC Yang Y Liao Z Zhao Y. From pre-diabetes to diabetes: diagnosis, treatments and translational research. Medicina (Kaunas). 2019;55:546. 31470636 10.3390/medicina55090546 PMC6780236

[16] Nathan DM Davidson MB DeFronzo RA. Impaired fasting glucose and impaired glucose tolerance: implications for care. Diabetes Care. 2007;30:753–9. 17327355 10.2337/dc07-9920

[17] Ramachandran A. Know the signs and symptoms of diabetes. Indian J Med Res. 2014;140:579–81. 25579136 PMC4311308

[18] Li Y Liu Y Liu S. Diabetic vascular diseases: molecular mechanisms and therapeutic strategies. Signal Transduct Target Ther. 2023;8:152. 37037849 10.1038/s41392-023-01400-z PMC10086073

[19] Horton WB Barrett EJ. Microvascular dysfunction in diabetes mellitus and cardiometabolic disease. Endocr Rev. 2021;42:29–55. 33125468 10.1210/endrev/bnaa025 PMC7846151

[20] Cohen K Shinkazh N Frank J Israel I Fellner C. Pharmacological treatment of diabetic peripheral neuropathy. P T. 2015;40:372–88. 26045647 PMC4450668

[21] Paul SK Klein K Thorsted BL Wolden ML Khunti K. Delay in treatment intensification increases the risks of cardiovascular events in patients with type 2 diabetes. Cardiovasc Diabetol. 2015;14:100. 26249018 10.1186/s12933-015-0260-x PMC4528846

[22] Davies MJ D’Alessio DA Fradkin J. Management of hyperglycemia in type 2 diabetes, 2018. A consensus report by the American Diabetes Association (ADA) and the European Association for the Study of Diabetes (EASD). Diabetes Care. 2018;41:2669–701. 30291106 10.2337/dci18-0033 PMC6245208

[23] Palmer M Sutherland J Barnard S. The effectiveness of smoking cessation, physical activity/diet and alcohol reduction interventions delivered by mobile phones for the prevention of non-communicable diseases: a systematic review of randomised controlled trials. PLoS One. 2018;13:e0189801. 29304148 10.1371/journal.pone.0189801 PMC5755775

[24] Dahlén AD Dashi G Maslov I. Trends in antidiabetic drug discovery: FDA approved drugs, new drugs in clinical trials and global sales. Front Pharmacol. 2022;12:807548. 35126141 10.3389/fphar.2021.807548 PMC8807560

[25] Filippatos TD Panagiotopoulou TV Elisaf MS. Adverse effects of GLP-1 receptor agonists. Rev Diabet Stud. 2014;11:202–30. 26177483 10.1900/RDS.2014.11.202 PMC5397288

[26] Yau JW Thor SM Ramadas A. Nutritional strategies in prediabetes: a scoping review of recent evidence. Nutrients. 2020;12:2990. 33003593 10.3390/nu12102990 PMC7650618

[27] van Dijk JW van Loon LJ. Exercise strategies to optimize glycemic control in type 2 diabetes: a continuing glucose monitoring perspective. Diabetes Spectr. 2015;28:24–31. 25717275 10.2337/diaspect.28.1.24 PMC4334084

[28] Kim SH Choi D. NaturaPredicta™: NLP-based functional scoring method for predicting the bioactivity and similarity of botanical ingredients. Food Suppl Biomater Health. 2023;3:e17.

[29] Alkhatib A Tsang C Tiss A. Functional foods and lifestyle approaches for diabetes prevention and management. Nutrients. 2017;9:1310. 29194424 10.3390/nu9121310 PMC5748760

[30] Samtiya M Aluko RE Dhewa T Moreno-Rojas JM. Potential health benefits of plant food-derived bioactive components: an overview. Foods. 2021;10:839. 33921351 10.3390/foods10040839 PMC8068854

[31] Lee HJ Lee YH Park SK. Korean red ginseng (Panax ginseng) improves insulin sensitivity and attenuates the development of diabetes in Otsuka Long-Evans Tokushima fatty rats. Metabolism. 2009;58:1170–7. 19477471 10.1016/j.metabol.2009.03.015

[32] Bang H Kwak JH Ahn HY Shin DY Lee JH. Korean red ginseng improves glucose control in subjects with impaired fasting glucose, impaired glucose tolerance, or newly diagnosed type 2 diabetes mellitus. J Med Food. 2014;17:128–34. 24456363 10.1089/jmf.2013.2889 PMC3901349

[33] Lee MS Lee MS Lim HJ Moon S-R. Survey of the use of complementary and alternative medicine among Korean diabetes mellitus patients. Pharmacoepidemiol Drug Saf. 2004;13:167–71. 15072116 10.1002/pds.877

[34] Oh MR Park SH Kim SY. Postprandial glucose-lowering effects of fermented red ginseng in subjects with impaired fasting glucose or type 2 diabetes: a randomized, double-blind, placebo-controlled clinical trial. BMC Complement Altern Med. 2014;14:237. 25015735 10.1186/1472-6882-14-237 PMC4227112

[35] Vuksan V Sung MK Sievenpiper JL. Korean red ginseng (Panax ginseng) improves glucose and insulin regulation in well-controlled, type 2 diabetes: results of a randomized, double-blind, placebo-controlled study of efficacy and safety. Nutr Metab Cardiovasc Dis. 2008;18:46–56. 16860976 10.1016/j.numecd.2006.04.003

[36] Kim JY Park JY Kang HJ Kim OY Lee JH. Beneficial effects of Korean red ginseng on lymphocyte DNA damage, antioxidant enzyme activity, and LDL oxidation in healthy participants: a randomized, double-blind, placebo-controlled trial. Nutr J. 2012;11:47. 22805313 10.1186/1475-2891-11-47 PMC3426460

[37] Seo SK Hong Y Yun BH. Antioxidative effects of Korean red ginseng in postmenopausal women: a double-blind randomized controlled trial. J Ethnopharmacol. 2014;154:753–7. 24814037 10.1016/j.jep.2014.04.051

[38] Lee YY Saba E Irfan M. The anti-inflammatory and anti-nociceptive effects of Korean black ginseng. Phytomedicine. 2019;54:169–81. 30668366 10.1016/j.phymed.2018.09.186

[39] Park JK Shim JY Cho AR Cho M-R Lee Y-J. Korean red ginseng protects against mitochondrial damage and intracellular inflammation in an animal model of type 2 diabetes mellitus. J Med Food. 2018;21:544–50. 29708804 10.1089/jmf.2017.4059

[40] Kang KS Yamabe N Kim HY Park JH Yokozawa T. Therapeutic potential of 20(S)-ginsenoside Rg(3) against streptozotocin-induced diabetic renal damage in rats. Eur J Pharmacol. 2008;591:266–72. 18611400 10.1016/j.ejphar.2008.06.077

[41] Kim YJ Zhang D Yang DC. Biosynthesis and biotechnological production of ginsenosides. Biotechnol Adv. 2015;33:717–35. 25747290 10.1016/j.biotechadv.2015.03.001

[42] Jeong S Yoon M. Fenofibrate inhibits adipocyte hypertrophy and insulin resistance by activating adipose PPARalpha in high fat diet-induced obese mice. Exp Mol Med. 2009;41:397–405. 19322024 10.3858/emm.2009.41.6.045 PMC2705860

[43] Lee JB Yoon SJ Lee SH. Ginsenoside Rg3 ameliorated HFD-induced hepatic steatosis through downregulation of STAT5-PPARγ. J Endocrinol. 2017;235:223–35. 29042402 10.1530/JOE-17-0233

[44] Hossain MA Lee D Kim B Kang C-W Kim NS Kim J-H. Korean red ginseng attenuates type 2 diabetic cardiovascular dysfunction in otsuka long-evans tokushima fatty rats. J Ginseng Res. 2020;44:308–11. 32148413 10.1016/j.jgr.2018.12.003 PMC7031734

[45] Ratnaningtyas NI Husen F Sukmawati D Wibowo ES Hikam AR Aksoy A. Antidiabetic effects and enzymatic antioxidant activity of chicken drumstick mushroom (coprinus comatus) extract in diabetic rats model. J Pure Appl Microbiol. 2022;16:2764–74.

[46] Shukla A Srinivasan BP. 16,17-Dihydro-17b-hydroxy isomitraphylline alkaloid as an inhibitor of DPP-IV, and its effect on incretin hormone and β-cell proliferation in diabetic rat. Eur J Pharm Sci. 2012;47:512–9. 22820565 10.1016/j.ejps.2012.07.012

[47] Adhikari B. Roles of alkaloids from medicinal plants in the management of diabetes mellitus. J Chem. 2021;2021:1–10.

[48] Cheng A Kessler D Mackinnon R. Conducting multicenter research in healthcare simulation: lessons learned from the INSPIRE network. Adv Simul (Lond). 2017;2:6. 29450007 10.1186/s41077-017-0039-0 PMC5806260

[49] Chung KC Song JW Group WS. A guide to organizing a multicenter clinical trial. Plast Reconstr Surg. 2010;126:515–23. 20375760 10.1097/PRS.0b013e3181df64fa PMC2917608

[50] Schwartz A Young R Hicks PJ; Appd Learn. Medical education practice-based research networks: facilitating collaborative research. Med Teach. 2016;38:64–74. 25319404 10.3109/0142159X.2014.970991 PMC4776698

[51] Ban JY Kang SW Lee JS Chung J-H Ko YG Choi HS. Korean red ginseng protects against neuronal damage induced by transient focal ischemia in rats. Exp Ther Med. 2012;3:693–8. 22969953 10.3892/etm.2012.449 PMC3438670

[52] Quan HY Kim DY Chung SH. Korean red ginseng extract alleviates advanced glycation end product-mediated renal injury. J Ginseng Res. 2013;37:187–93. 23717171 10.5142/jgr.2013.37.187 PMC3659634

[53] Sen S Chen S Wu Y Feng B Lui EK Chakrabarti S. Preventive effects of North American ginseng (Panax quinquefolius) on diabetic retinopathy and cardiomyopathy. Phytother Res. 2013;27:290–8. 22566158 10.1002/ptr.4719

